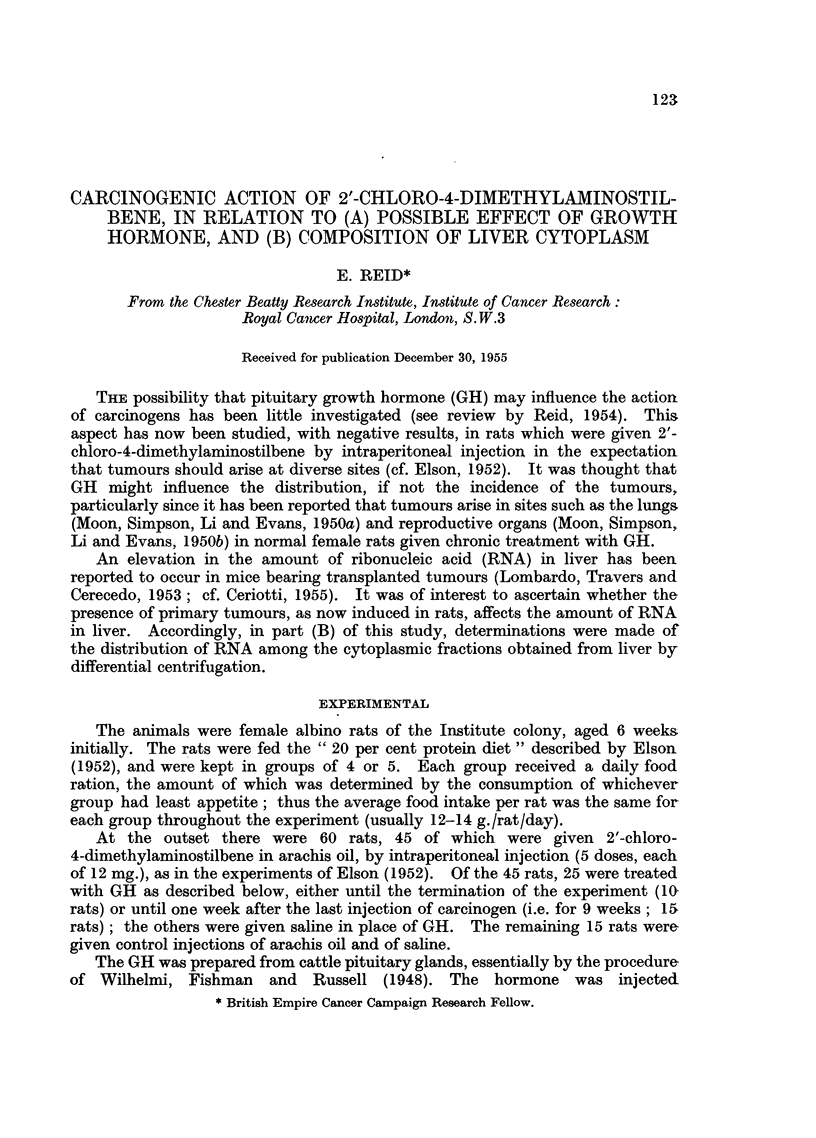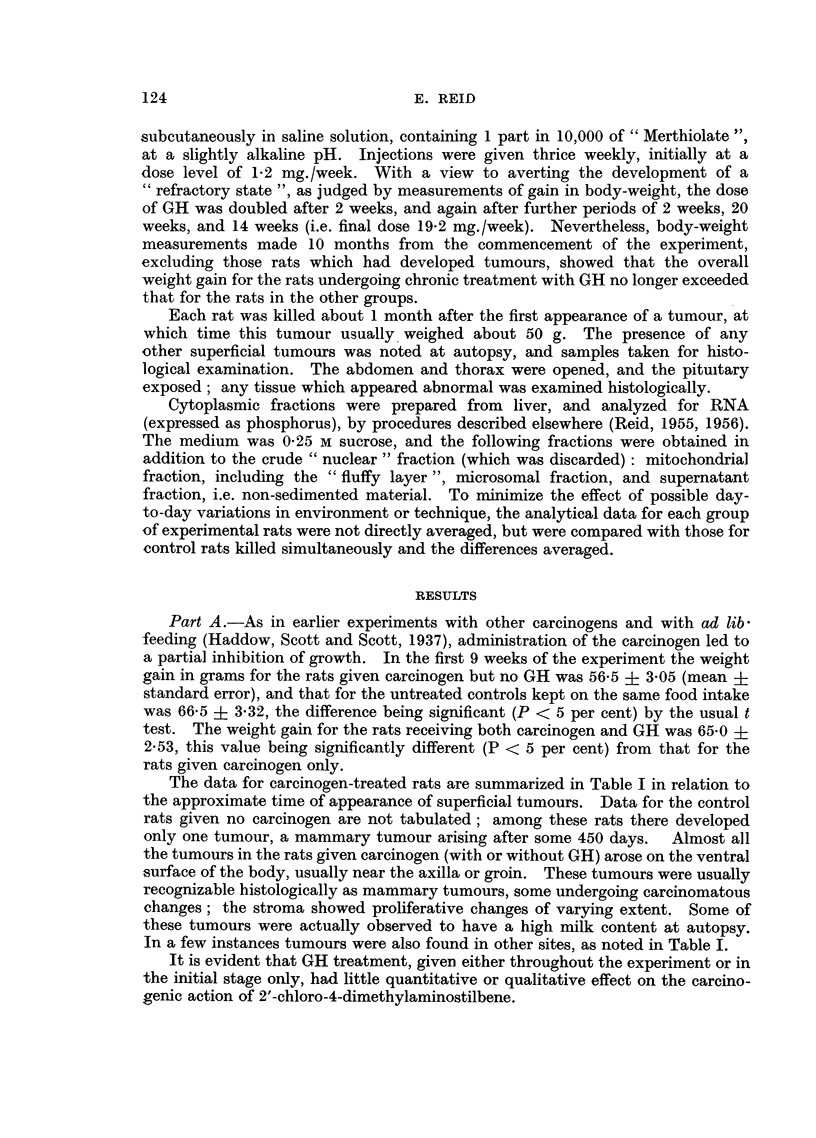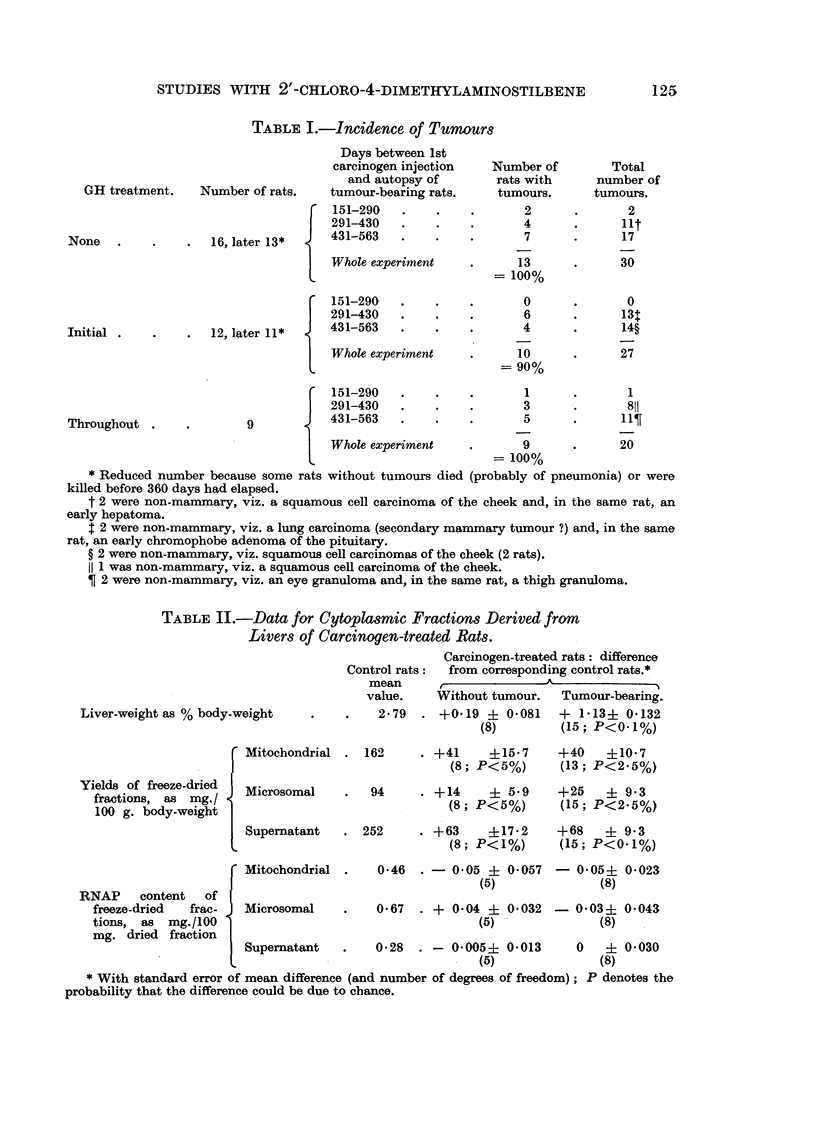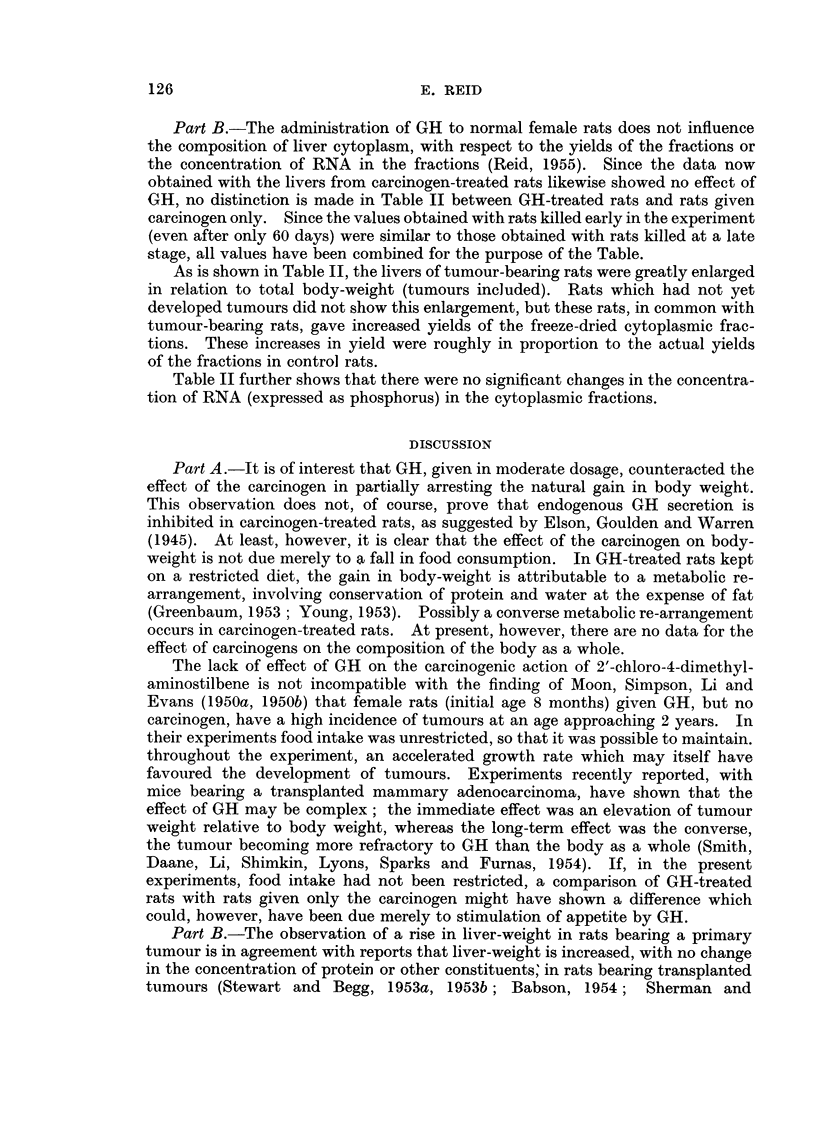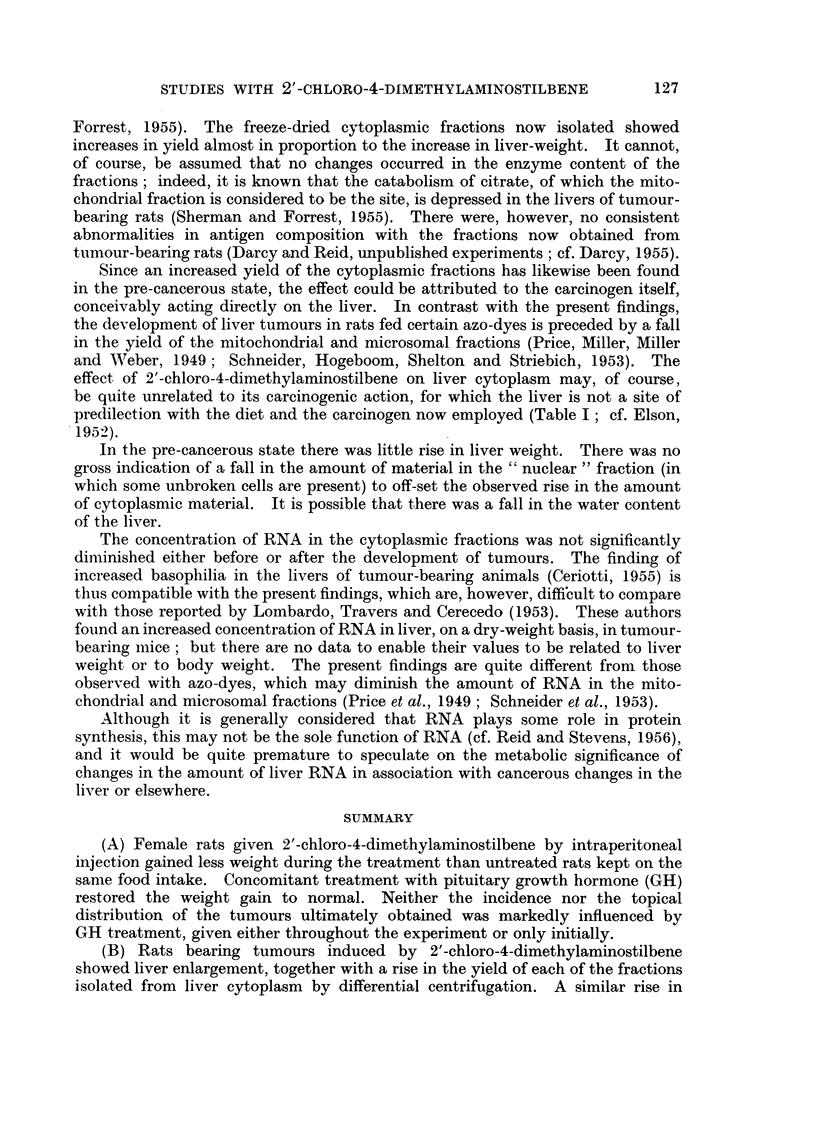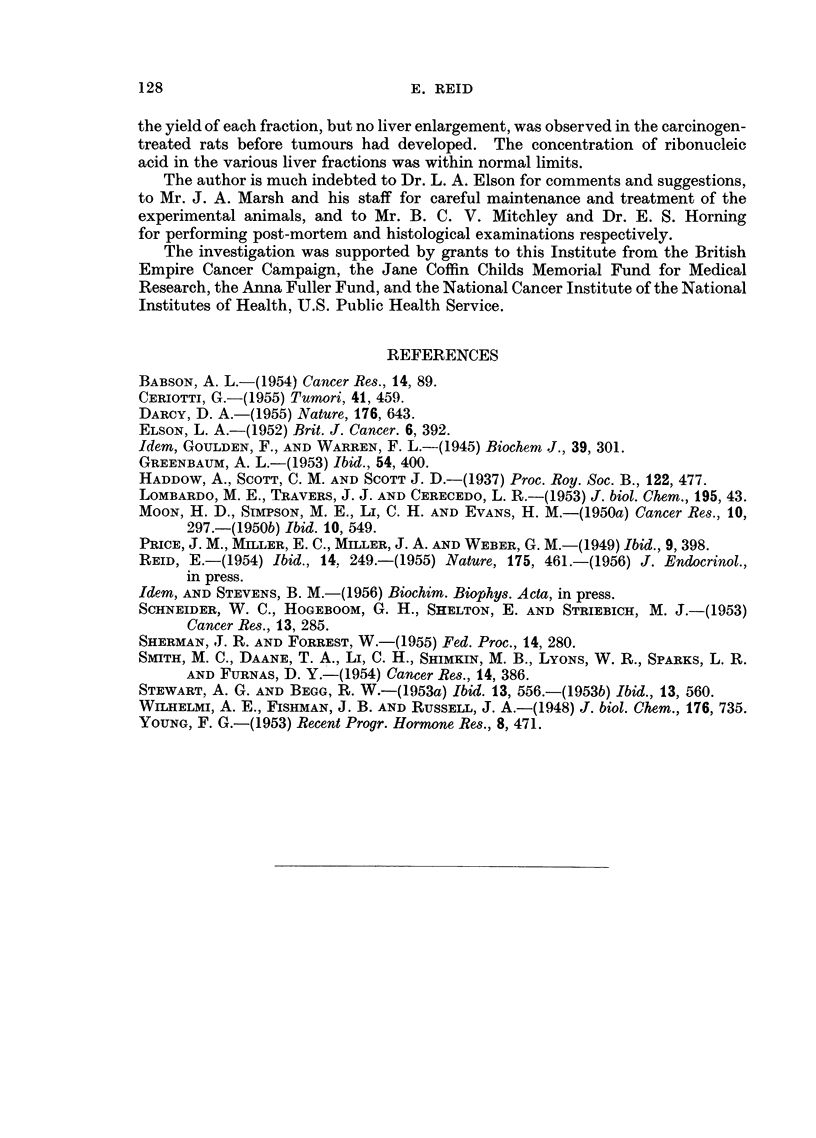# Carcinogenic Action of 2′-Chloro-4-Dimethylaminostilbene, in Relation to (A) Possible Effect of Growth Hormone, and (B) Composition of Liver Cytoplasm

**DOI:** 10.1038/bjc.1956.16

**Published:** 1956-03

**Authors:** E. Reid


### CARCINOGENIC ACTION OF 2′-CHLORO-4-DIMETHYLAMINOSTILBENE, IN RELATION TO (A) POSSIBLE EFFECT OF GROWTH HORMONE, AND (B) COMPOSITION OF LIVER CYTOPLASM

E. REID*

*From the Chester Beatty Research Institute, Institute of Cancer Research : Royal Cancer Hospital, London, S.W.3*

Received for publication December 30, 1955

THE possibility that pituitary growth hormone (GH) may influence the action of carcinogens has been little investigated (see review by Reid, 1954). This aspect has now been studied, with negative results, in rats which were given 2′-chloro-4-dimethylaminostilbene by intraperitoneal injection in the expectation that tumours should arise at diverse sites (cf. Elson, 1952). It was thought that GH might influence the distribution, if not the incidence of the tumours, particularly since it has been reported that tumours arise in sites such as the lungs (Moon, Simpson, Li and Evans, 1950*a*) and reproductive organs (Moon, Simpson, Li and Evans, 1950*b*) in normal female rats given chronic treatment with GH.

An elevation in the amount of ribonucleic acid (RNA) in liver has been reported to occur in mice bearing transplanted tumours (Lombardo, Travers and Cerecedo, 1953 ; cf. Ceriotti, 1955). It was of interest to ascertain whether the presence of primary tumours, as now induced in rats, affects the amount of RNA in liver. Accordingly, in part (B) of this study, determinations were made of the distribution of RNA among the cytoplasmic fractions obtained from liver by differential centrifugation.

### EXPERIMENTAL

The animals were female albino rats of the Institute colony, aged 6 weeks initially. The rats were fed the " 20 per cent protein diet " described by Elson (1952), and were kept in groups of 4 or 5. Each group received a daily food ration, the amount of which was determined by the consumption of whichever group had least appetite ; thus the average food intake per rat was the same for each group throughout the experiment (usually 12–14 g./rat/day).

At the outset there were 60 rats, 45 of which were given 2′-chloro-4-dimethylaminostilbene in arachis oil, by intraperitoneal injection (5 doses, each of 12 mg.), as in the experiments of Elson (1952). Of the 45 rats, 25 were treated with GH as described below, either until the termination of the experiment (10 rats) or until one week after the last injection of carcinogen (i.e. for 9 weeks ; 15 rats) ; the others were given saline in place of GH. The remaining 15 rats were given control injections of arachis oil and of saline.

The GH was prepared from cattle pituitary glands, essentially by the procedure of Wilhelmi, Fishman and Russell (1948). The hormone was injected

* British Empire Cancer Campaign Research Fellow.

subcutaneously in saline solution, containing 1 part in 10,000 of " Merthiolate ", at a slightly alkaline pH. Injections were given thrice weekly, initially at a dose level of 1·2 mg./week. With a view to averting the development of a " refractory state ", as judged by measurements of gain in body-weight, the dose of GH was doubled after 2 weeks, and again after further periods of 2 weeks, 20 weeks, and 14 weeks (i.e. final dose 19·2 mg./week). Nevertheless, body-weight measurements made 10 months from the commencement of the experiment, excluding those rats which had developed tumours, showed that the overall weight gain for the rats undergoing chronic treatment with GH no longer exceeded that for the rats in the other groups.

Each rat was killed about 1 month after the first appearance of a tumour, at which time this tumour usually weighed about 50 g. The presence of any other superficial tumours was noted at autopsy, and samples taken for histological examination. The abdomen and thorax were opened, and the pituitary exposed ; any tissue which appeared abnormal was examined histologically.

Cytoplasmic fractions were prepared from liver, and analyzed for RNA (expressed as phosphorus), by procedures described elsewhere (Reid, 1955, 1956). The medium was 0·25 M sucrose, and the following fractions were obtained in addition to the crude " nuclear " fraction (which was discarded) : mitochondrial fraction, including the " fluffy layer ", microsomal fraction, and supernatant fraction, i.e. non-sedimented material. To minimize the effect of possible day-to-day variations in environment or technique, the analytical data for each group of experimental rats were not directly averaged, but were compared with those for control rats killed simultaneously and the differences averaged.

### RESULTS

*Part A.*—As in earlier experiments with other carcinogens and with *ad lib* feeding (Haddow, Scott and Scott, 1937), administration of the carcinogen led to a partial inhibition of growth. In the first 9 weeks of the experiment the weight gain in grams for the rats given carcinogen but no GH was 56·5 $\pm$ 3·05 (mean $\pm$ standard error), and that for the untreated controls kept on the same food intake was 66·5 $\pm$ 3·32, the difference being significant ($P < 5$ per cent) by the usual $t$ test. The weight gain for the rats receiving both carcinogen and GH was 65·0 $\pm$ 2·53, this value being significantly different (P $<$ 5 per cent) from that for the rats given carcinogen only.

The data for carcinogen-treated rats are summarized in Table I in relation to the approximate time of appearance of superficial tumours. Data for the control rats given no carcinogen are not tabulated ; among these rats there developed only one tumour, a mammary tumour arising after some 450 days. Almost all the tumours in the rats given carcinogen (with or without GH) arose on the ventral surface of the body, usually near the axilla or groin. These tumours were usually recognizable histologically as mammary tumours, some undergoing carcinomatous changes ; the stroma showed proliferative changes of varying extent. Some of these tumours were actually observed to have a high milk content at autopsy. In a few instances tumours were also found in other sites, as noted in Table I.

It is evident that GH treatment, given either throughout the experiment or in the initial stage only, had little quantitative or qualitative effect on the carcinogenic action of 2′-chloro-4-dimethylaminostilbene.

TABLE I.—*Incidence of Tumours*

| GH treatment. | Number of rats. | Days between 1st carcinogen injection and autopsy of tumour-bearing rats. | Number of rats with tumours. | Total number of tumours. |
| --- | --- | --- | --- | --- |
| None | 16, later 13* | 151–290 | 2 | 2 |
| | | 291–430 | 4 | 11† |
| | | 431–563 | 7 | 17 |
| | | *Whole experiment* | 13 = 100% | 30 |
| Initial | 12, later 11* | 151–290 | 0 | 0 |
| | | 291–430 | 6 | 13‡ |
| | | 431–563 | 4 | 14§ |
| | | *Whole experiment* | 10 = 90% | 27 |
| Throughout | 9 | 151–290 | 1 | 1 |
| | | 291–430 | 3 | 8‖ |
| | | 431–563 | 5 | 11¶ |
| | | *Whole experiment* | 9 = 100% | 20 |

* Reduced number because some rats without tumours died (probably of pneumonia) or were killed before 360 days had elapsed.

† 2 were non-mammary, viz. a squamous cell carcinoma of the cheek and, in the same rat, an early hepatoma.

‡ 2 were non-mammary, viz. a lung carcinoma (secondary mammary tumour ?) and, in the same rat, an early chromophobe adenoma of the pituitary.

§ 2 were non-mammary, viz. squamous cell carcinomas of the cheek (2 rats).

‖ 1 was non-mammary, viz. a squamous cell carcinoma of the cheek.

¶ 2 were non-mammary, viz. an eye granuloma and, in the same rat, a thigh granuloma.

TABLE II.—*Data for Cytoplasmic Fractions Derived from Livers of Carcinogen-treated Rats.*

| | | Control rats : mean value. | Carcinogen-treated rats : difference from corresponding control rats.* Without tumour. | Tumour-bearing. |
| --- | --- | --- | --- | --- |
| Liver-weight as % body-weight | | 2·79 | +0·19 ± 0·081 (8) | + 1·13 ± 0·132 (15 ; $P<0·1\%$) |
| Yields of freeze-dried fractions, as mg./100 g. body-weight | Mitochondrial | 162 | +41 ±15·7 (8 ; $P<5\%$) | +40 ±10·7 (13 ; $P<2·5\%$) |
| | Microsomal | 94 | +14 ± 5·9 (8 ; $P<5\%$) | +25 ± 9·3 (15 ; $P<2·5\%$) |
| | Supernatant | 252 | +63 ±17·2 (8 ; $P<1\%$) | +68 ± 9·3 (15 ; $P<0·1\%$) |
| RNAP content of freeze-dried fractions, as mg./100 mg. dried fraction | Mitochondrial | 0·46 | — 0·05 ± 0·057 (5) | — 0·05 ± 0·023 (8) |
| | Microsomal | 0·67 | + 0·04 ± 0·032 (5) | — 0·03 ± 0·043 (8) |
| | Supernatant | 0·28 | — 0·005 ± 0·013 (5) | 0 ± 0·030 (8) |

* With standard error of mean difference (and number of degrees of freedom) ; $P$ denotes the probability that the difference could be due to chance.

*Part B.*—The administration of GH to normal female rats does not influence the composition of liver cytoplasm, with respect to the yields of the fractions or the concentration of RNA in the fractions (Reid, 1955). Since the data now obtained with the livers from carcinogen-treated rats likewise showed no effect of GH, no distinction is made in Table II between GH-treated rats and rats given carcinogen only. Since the values obtained with rats killed early in the experiment (even after only 60 days) were similar to those obtained with rats killed at a late stage, all values have been combined for the purpose of the Table.

As is shown in Table II, the livers of tumour-bearing rats were greatly enlarged in relation to total body-weight (tumours included). Rats which had not yet developed tumours did not show this enlargement, but these rats, in common with tumour-bearing rats, gave increased yields of the freeze-dried cytoplasmic fractions. These increases in yield were roughly in proportion to the actual yields of the fractions in control rats.

Table II further shows that there were no significant changes in the concentration of RNA (expressed as phosphorus) in the cytoplasmic fractions.

### DISCUSSION

*Part A.*—It is of interest that GH, given in moderate dosage, counteracted the effect of the carcinogen in partially arresting the natural gain in body weight. This observation does not, of course, prove that endogenous GH secretion is inhibited in carcinogen-treated rats, as suggested by Elson, Goulden and Warren (1945). At least, however, it is clear that the effect of the carcinogen on body-weight is not due merely to a fall in food consumption. In GH-treated rats kept on a restricted diet, the gain in body-weight is attributable to a metabolic re-arrangement, involving conservation of protein and water at the expense of fat (Greenbaum, 1953 ; Young, 1953). Possibly a converse metabolic re-arrangement occurs in carcinogen-treated rats. At present, however, there are no data for the effect of carcinogens on the composition of the body as a whole.

The lack of effect of GH on the carcinogenic action of 2′-chloro-4-dimethyl-aminostilbene is not incompatible with the finding of Moon, Simpson, Li and Evans (1950*a*, 1950*b*) that female rats (initial age 8 months) given GH, but no carcinogen, have a high incidence of tumours at an age approaching 2 years. In their experiments food intake was unrestricted, so that it was possible to maintain. throughout the experiment, an accelerated growth rate which may itself have favoured the development of tumours. Experiments recently reported, with mice bearing a transplanted mammary adenocarcinoma, have shown that the effect of GH may be complex ; the immediate effect was an elevation of tumour weight relative to body weight, whereas the long-term effect was the converse, the tumour becoming more refractory to GH than the body as a whole (Smith, Daane, Li, Shimkin, Lyons, Sparks and Furnas, 1954). If, in the present experiments, food intake had not been restricted, a comparison of GH-treated rats with rats given only the carcinogen might have shown a difference which could, however, have been due merely to stimulation of appetite by GH.

*Part B.*—The observation of a rise in liver-weight in rats bearing a primary tumour is in agreement with reports that liver-weight is increased, with no change in the concentration of protein or other constituents, in rats bearing transplanted tumours (Stewart and Begg, 1953*a*, 1953*b* ; Babson, 1954 ; Sherman and

Forrest, 1955). The freeze-dried cytoplasmic fractions now isolated showed increases in yield almost in proportion to the increase in liver-weight. It cannot, of course, be assumed that no changes occurred in the enzyme content of the fractions ; indeed, it is known that the catabolism of citrate, of which the mitochondrial fraction is considered to be the site, is depressed in the livers of tumour-bearing rats (Sherman and Forrest, 1955). There were, however, no consistent abnormalities in antigen composition with the fractions now obtained from tumour-bearing rats (Darcy and Reid, unpublished experiments ; cf. Darcy, 1955).

Since an increased yield of the cytoplasmic fractions has likewise been found in the pre-cancerous state, the effect could be attributed to the carcinogen itself, conceivably acting directly on the liver. In contrast with the present findings, the development of liver tumours in rats fed certain azo-dyes is preceded by a fall in the yield of the mitochondrial and microsomal fractions (Price, Miller, Miller and Weber, 1949 ; Schneider, Hogeboom, Shelton and Striebich, 1953). The effect of 2′-chloro-4-dimethylaminostilbene on liver cytoplasm may, of course, be quite unrelated to its carcinogenic action, for which the liver is not a site of predilection with the diet and the carcinogen now employed (Table I ; cf. Elson, 1952).

In the pre-cancerous state there was little rise in liver weight. There was no gross indication of a fall in the amount of material in the " nuclear " fraction (in which some unbroken cells are present) to off-set the observed rise in the amount of cytoplasmic material. It is possible that there was a fall in the water content of the liver.

The concentration of RNA in the cytoplasmic fractions was not significantly diminished either before or after the development of tumours. The finding of increased basophilia in the livers of tumour-bearing animals (Ceriotti, 1955) is thus compatible with the present findings, which are, however, difficult to compare with those reported by Lombardo, Travers and Cerecedo (1953). These authors found an increased concentration of RNA in liver, on a dry-weight basis, in tumour-bearing mice ; but there are no data to enable their values to be related to liver weight or to body weight. The present findings are quite different from those observed with azo-dyes, which may diminish the amount of RNA in the mitochondrial and microsomal fractions (Price *et al.*, 1949 ; Schneider *et al.*, 1953).

Although it is generally considered that RNA plays some role in protein synthesis, this may not be the sole function of RNA (cf. Reid and Stevens, 1956), and it would be quite premature to speculate on the metabolic significance of changes in the amount of liver RNA in association with cancerous changes in the liver or elsewhere.

### SUMMARY

(A) Female rats given 2′-chloro-4-dimethylaminostilbene by intraperitoneal injection gained less weight during the treatment than untreated rats kept on the same food intake. Concomitant treatment with pituitary growth hormone (GH) restored the weight gain to normal. Neither the incidence nor the topical distribution of the tumours ultimately obtained was markedly influenced by GH treatment, given either throughout the experiment or only initially.

(B) Rats bearing tumours induced by 2′-chloro-4-dimethylaminostilbene showed liver enlargement, together with a rise in the yield of each of the fractions isolated from liver cytoplasm by differential centrifugation. A similar rise in the yield of each fraction, but no liver enlargement, was observed in the carcinogen-treated rats before tumours had developed. The concentration of ribonucleic acid in the various liver fractions was within normal limits.

The author is much indebted to Dr. L. A. Elson for comments and suggestions, to Mr. J. A. Marsh and his staff for careful maintenance and treatment of the experimental animals, and to Mr. B. C. V. Mitchley and Dr. E. S. Horning for performing post-mortem and histological examinations respectively.

The investigation was supported by grants to this Institute from the British Empire Cancer Campaign, the Jane Coffin Childs Memorial Fund for Medical Research, the Anna Fuller Fund, and the National Cancer Institute of the National Institutes of Health, U.S. Public Health Service.

### REFERENCES

BABSON, A. L.—(1954) *Cancer Res.*, **14**, 89.
CERIOTTI, G.—(1955) *Tumori*, **41**, 459.
DARCY, D. A.—(1955) *Nature*, **176**, 643.
ELSON, L. A.—(1952) *Brit. J. Cancer.* **6**, 392.
*Idem*, GOULDEN, F., AND WARREN, F. L.—(1945) *Biochem J.*, **39**, 301.
GREENBAUM, A. L.—(1953) *Ibid.*, **54**, 400.
HADDOW, A., SCOTT, C. M. AND SCOTT J. D.—(1937) *Proc. Roy. Soc.* B., **122**, 477.
LOMBARDO, M. E., TRAVERS, J. J. AND CERECEDO, L. R.—(1953) *J. biol. Chem.*, **195**, 43.
MOON, H. D., SIMPSON, M. E., LI, C. H. AND EVANS, H. M.—(1950*a*) *Cancer Res.*, **10**, 297.—(1950*b*) *Ibid.* **10**, 549.
PRICE, J. M., MILLER, E. C., MILLER, J. A. AND WEBER, G. M.—(1949) *Ibid.*, **9**, 398.
REID, E.—(1954) *Ibid.*, **14**, 249.—(1955) *Nature*, **175**, 461.—(1956) *J. Endocrinol.*, in press.
*Idem*, AND STEVENS, B. M.—(1956) *Biochim. Biophys. Acta*, in press.
SCHNEIDER, W. C., HOGEBOOM, G. H., SHELTON, E. AND STRIEBICH, M. J.—(1953) *Cancer Res.*, **13**, 285.
SHERMAN, J. R. AND FORREST, W.—(1955) *Fed. Proc.*, **14**, 280.
SMITH, M. C., DAANE, T. A., LI, C. H., SHIMKIN, M. B., LYONS, W. R., SPARKS, L. R. AND FURNAS, D. Y.—(1954) *Cancer Res.*, **14**, 386.
STEWART, A. G. AND BEGG, R. W.—(1953*a*) *Ibid.* **13**, 556.—(1953*b*) *Ibid.*, **13**, 560.
WILHELMI, A. E., FISHMAN, J. B. AND RUSSELL, J. A.—(1948) *J. biol. Chem.*, **176**, 735.
YOUNG, F. G.—(1953) *Recent Progr. Hormone Res.*, **8**, 471.